# Factors associated with knowledge about malaria prevention among women of reproductive age, Tete Province, Mozambique, 2019–2020

**DOI:** 10.1186/s12936-022-04090-0

**Published:** 2022-03-05

**Authors:** Gerson Afai, Erika Valeska Rossetto, Cynthia Semá Baltazar, Baltazar Candrinho, Abuchahama Saifodine, Rose Zulliger

**Affiliations:** 1 Mozambique Field Epidemiology Training Programme, Instituto Nacional de Saúde, P.O. Box 264, Maputo, Mozambique; 2 MassGenics, Assigned for Centers for Disease Control and Prevention, Maputo, Mozambique; 3 National Institute of Health, Maputo, Mozambique; 4 National Malaria Control Programme, Ministry of Health, Maputo, Mozambique; 5 United States President’s Malaria Initiative, USAID, Maputo, Mozambique; 6 United States President’s Malaria Initiative, USAID, Washington, D.C. USA

**Keywords:** Malaria, Health Knowledge, Risk Factors, Mozambique

## Abstract

**Background:**

Mozambique is a malaria endemic country with an estimated prevalence of malaria in children 6–59 months old that is twice as high in rural areas (46.0%) as in urban areas (18.0%). However, only 46.0% of women aged 15–49 years had complete knowledge about malaria in 2018. This study aimed to identify the factors associated with malaria knowledge among women of reproductive age in a high malaria burden district.

**Methods:**

Data from a cross-sectional study, using a population-based malaria research study in Mágoe District, 2019, were analysed. This analysis included women aged 15–49 years. A multivariate logistic regression model was developed to determine factors associated with complete knowledge of malaria that calculated adjusted odds ratio (aOR) and 95% confidence interval (CI) at a p < 0.05 significance level. Complete malaria knowledge was defined as when a woman correctly identified: fever as a malaria symptom, mosquito bites as the means of malaria transmission, mosquito nets as a tool for malaria prevention, malaria as curable, and were able to name an anti-malarial.

**Results:**

A total of 1899 women were included in this analysis. There was complete malaria knowledge among 49% of the respondents. Seventy one percent mentioned fever as one of malaria symptoms, 92% mentioned mosquito bite as the cause of malaria infection, 94% identified that mosquito nets prevent malaria, 92% agreed that malaria has cure, and 76% were able to name at least one anti-malarial medicine. In the multivariate analysis, the following characteristics were associated with significantly higher odds of having complete malaria knowledge: having a secondary school or above education level (adjusted Odds Ratio, aOR = 2.5 CI [1.3–4.6] p = 0.005), being from the middle socioeconomic status group (aOR = 1.5 CI [1.1–2.1] p = 0.005), being from older age group of 35–39 (aOR = 1.9; CI [1.1–3.1] p < 0.001), having 1–2 children (aOR = 1.8; CI [1.2–2.6] p = 0.003), and having interviews completed in Portuguese or Cinyungwe (aOR = 2.3; CI [1.3–4.1] p = 0.004 and aOR = 2.1; CI [1.5–2.8] p < 0.001, respectively).

**Conclusion:**

Most women in this study had some malaria knowledge, but gaps in complete knowledge remained. In order to broaden knowledge, educational messages about malaria prevention should be more effectively targeted to reach younger, less-educated women and in non-dominant languages.

## Background

Worldwide, there has been remarkable progress in reducing malaria cases and deaths over the past decades as shown by the decrease from 238 million global cases in 2000 to 228 million cases in 2019 [1]. This reduction in transmission has been accompanied by a decrease in deaths from 736,000 in 2000 to 409,000 in 2019 [1]. Despite this progress, the World Health Organization (WHO) African Region continues to have the highest global malaria burden, representing 94.0% of global cases, and Mozambique is among the top five countries with 5.0% of the global malaria burden [1].

According to the 2020 NMCP report, the trend of malaria cases reported by health facilities and community health workers was to increase by 4.0%, from the previous 10,864,677 in 2019, to 11,331,009 in 2020. In Tete province, the trend of cases was also increased by 12.0%, from 725,745 in 2019, to 812,239 in 2020. Regarding mortality, the trend was decreasing, from 1.4 deaths per 100,000 inhabitants in 2019 to 1.0 deaths per 100,000 inhabitants in 2020 [2].

The WHO recommends implementing malaria prevention strategies through vector control and chemoprevention in certain population groups [3]. In Mozambique, the primary malaria prevention strategy is vector control through universal coverage campaigns of insecticide-treated nets (ITN) and targeted indoor residual spraying (IRS).

Women of reproductive age, who are pregnant, are a high-risk group for malaria infection, and the consequences are maternal anaemia, placental parasitaemia, retardation of intrauterine growth of the fetus, preterm birth, and low birth weight [4]. So, for pregnant women, in addition to offering ITN at antenatal care (ANC) to maintain universal coverage, intermittent preventive treatment in pregnancy with sulfadoxine-pyrimethamine (IPTp-SP) is also administered [5, 6].

According to the Mozambique Malaria Indicators Survey (MIS) from 2018, with the exception of Nampula province, less than half of women aged 15–49 years old had complete knowledge about the cause, symptoms, prevention and treatment of malaria. In Tete Province, only 46.0% of women had complete knowledge. Knowledge gaps existed between urban and rural with 87.0% of women in urban areas correctly reporting that sleeping under a mosquito net can protect against malaria, compared to 77.0% in rural areas. In the same survey, the number of women who received more than four doses of IPTp-SP was 30.8% in urban areas compared to 17.6% in rural areas, indicative of important differences in malaria knowledge and service access across urban/rural zones [6].

Individual factors, such as age, education level, occupation, wealth index, may influence malaria knowledge [7–13]. Knowledge of the sociocultural aspects of communities is a key factor in the success of malaria prevention strategies and has been a major challenge of malaria control programmes which seek to improve communication strategies for behaviour change [6]. Knowledge about malaria by women can contribute to they take decisions about their own and children's health care seeking [14]. Stronger insights into factors associated with malaria knowledge, attitudes and practices is relevant for Mozambique to achieve the social and behaviour change (SBC) goal of the Malaria Strategic Plan: which is to ensure that 70.0% of people seek adequate and timely health care and at least 85.0% of the population uses an appropriate protection method by 2022 [5].

Evidence-based decision-making is one of the strategies of the Mozambique National Malaria Control Programme, yet there are few studies conducted to understand the level and the factors influencing knowledge about malaria, particularly in rural areas. To appropriately target messages and stratify populations in order to prevent malaria in women of reproductive age, it is necessary to identify the factors associated with knowledge about malaria, its cause, symptoms and prevention. This study aimed to assess factors associated with knowledge about malaria among women of reproductive age in Mágoe district, Tete province, in 2019.

## Methods

### Study design and setting

This analysis used data from a cross-sectional study that was intended to serve as the baseline of the study on "cost-effectiveness of malaria communication strategies on social and behavioural change", conducted in November 2019 in Mágoe district, Tete province in Mozambique. Magoe District has a population of 89,273 inhabitants, with a density population of 10.3 inhabitants per square kilometre, and is located in the southwest of Tete province, bordered to the north by Zumbo and Maravia districts, to the east and south by the Republic of Zimbabwe, and to the east with Cahora Bassa District [15, 16]. Two administrative posts were included for the study, Mphende and Mucumbura. Mphende is the headquarters of the Mágoe district, and Mucumbura is located near the border with the Republic of Zimbabwe. The health services of Mucumbura, attend besides the population of Mozambique, the population of Zimbabwe. Mágoe district was selected because it had high malaria incidence with 27 cases per 1,000 habitants in the year 2019, has high community radio coverage, and was not covered by interpersonal communication malaria SBC interventions at the time of data collection.

### Data collection

Two structured questionnaires, a household questionnaire and a questionnaire for women of reproductive age (15–49 years old) were utilized. These questionnaires were based on the Malaria Behaviour Survey and MIS questionnaires and were tested in a rural area of Tete City to validate the questions and ensure its functionality and appropriateness for the local context. The questionnaires included questions related to socio-economic indicators, possession of ITN, history of fever in the past 2 weeks, history of recent travel, and current pregnancy, along with extensive questions on ideational factors associated with each key malaria behaviour (e.g. self-efficacy to use an ITN). Before the research began the interviewers were trained on the methods of obtaining consent and conducting the interviews. The interviewers were individuals with knowledge of the Cinyungwe, Shona, Tawala and Portuguese languages, as these are the most widely spoken languages in the region.

All enumeration areas in Mágoe District were eligible to be randomly selected for the study. Pre-existing lists of all enumeration areas (EA), including population size estimates and maps from Mozambique's National Institute of Statistics were used to develop a list of the EA to be selected for inclusion in the study. A sub-group of EA was selected in a manner designed to minimize bias by ensuring as much geographic separation of the EA as possible. Once selected, 120 EA were visited and fully enumerated. In each EA, 15 households were randomly selected. Households eligible for the study had to have one child under five years old and at least one mosquito net for every two residents under 5 years old.

### Study variables

In this study, the following socio-demographic variables were analyzed: age (15–19, 20–24, 25–29, 30–34, 35–39, 40–44 and 45–49), place of residence (Mphende Locality /Mucumburra Locality), marital status (single/married), education level (none, primary, complete primary, secondary, secondary completed and above), literacy (does not read, read part of a sentence, read a whole sentence), number of births (none, 1 to 2, 3 to 4, 5 and more births), language used for the interview (Portuguese, English, Cinyungwe, Cyiau, other), exposure to malaria messages in the six months before the survey (yes/no) and wealth quintile (lowest, second lowest, middle, second highest, highest).

The variable complete knowledge about malaria was a composite variable composed of the following five variables: signs and symptoms of malaria, mode of transmission, prevention, treatment, and whether malaria is curable. For signs and symptoms, the woman had to answer fever as a symptom of malaria, for mode of transmission she had to answer mosquito bite, for prevention she had to refer to mosquito net treated or not with insecticide, and for malaria treatment the women had to indicate at least one antimalarial drug in order to be categorized as having complete malaria knowledge.

### Data analysis

Weighted analyses were performed in the statistical program *STATA 16.1 (StataCorp LLC., College Station, Texas)*. Sociodemographic and other characteristics were presented as percentages and frequencies. The assessment of factors associated with knowledge about malaria was through logistic regression and knowledge about malaria was considered as dependent variable. To measure complete knowledge about malaria, in yes/no, the woman had to answer five questions about malaria. If she answered all five questions correctly, she was considered to have complete knowledge about malaria, otherwise, she was not considered to have complete knowledge. Variables that were statistically significant (p-value < 0.05) in the bivariate logistic regression analysis at 95.0% confidence interval (CI) or considered important a priori based on existent literature, were included in the multivariate regression to calculate the adjusted odds ratio (aOR).

## Results

### Characteristics of the participants

Table 1 illustrates the sociodemographic characteristics of the women participating in the study. A total of 1923 women were interviewed in the study, of whom 24 were excluded because where aged equal or more than 50 years, and 1899 women aged 15–49 years were eligible to the analysis, 68.0% were from Mucumburra. The mean age of the women was 27 (SD ± 8.4) years. The highest proportion of women was 15–19 years old, at 24.4% of the sample. Regarding marital status, 76.4% lived with a partner in their residence, and the rest were single. A total of 40.7% of participants had a primary school level education and 19.4% had no schooling. Only 31.8% were literate. Household ownership of goods that allow access to information was 32.6% for radio and 18.5% for television. Of all women, 86.5% had not heard or seen a message about malaria in the last six months before the interview. Regarding the number of births, the women have had in their lifetime, 30.5% had between one and two births, and 14.7% had no births. Of the total number of women surveyed, 83.5% of interviews were conducted in the Cinyungwe language, and 4.7% were done using Portuguese. A total of 22.5% of women were in households from the lowest quintile.

**Table 1:** Distribution of sociodemographic and other characteristics of women of reproductive age in Magoe, Tete, 2019

Characteristics	N = 1899	%
Group age		
Mean (SD), in years	27 (8.4)	
15–19	463	24.38
20–24	436	22.96
25–29	359	18.90
30–34	264	13.90
25–39	204	10.74
40–44	114	6.00
45–49	59	3.11
Marital status		
Single	448	23.59
Living with partner	1451	76.41
Place of residence		
Mphende	605	31.86
Mucumburra	1294	68.14
Level school		
None	369	19.43
Primary incomplete	772	40.65
Primary complete	336	17.69
Secondary	331	17.43
Secondary complete or above	91	4.79
Literacy		
Does not read at all	795	42.11
Read part of a sentence	493	26.11
Read a whole sentence	600	31.78
Number of Births		
None	279	14.69
1 to 2 children	674	35.49
3 to 4 children	570	30.02
5 and more children	376	19.80
Radio ownership		
No	1280	67.40
Yes	619	32.60
Television ownership		
No	1548	81.52
Yes	351	18.48
Exposure to malaria message in the last 6 months		
No	1642	86.47
Yes	257	13.53
Wealth quintile		
Lower	428	22.54
Second	334	17.59
Medium	408	21.48
Fourth	336	17.69
Highest	393	20.70

### Distribution of knowledge of causes, symptoms, prevention and cure of malaria

Table 2 illustrates that of all participants, 70.9% recognized fever as a symptom of malaria, 91.8% knew that a mosquito bite is the way malaria is transmitted, and 93.9% mentioned a mosquito net as the way to prevent malaria. A total of 91.5% knew that malaria is curable, however, 76.4% did not know of any anti-malarials. Significant differences in knowledge by administrative post existed for all questions, but did not exist for the complete knowledge.

**Table 2:** Participant’s malaria knowledge causes, symptoms, prevention and cure by place of residence, Magoe, Tete, 2019

Knowledge	Total		Residence				χ^2^	p-value
	N = 1899	%	Mphende		Mucumburra			
			n = 605	%	n = 1294	%		
Fever is malaria symptom								
No	553	29.12	231	38.18	322	24.88	35.32	< 0.001
Yes	1346	70.88	374	61.82	972	75.12		
Mosquito bites transmit malaria								
No	155	8.16	62	10.25	93	7.19	5.15	0.023
Yes	1744	91.84	543	89.75	1201	92.81		
Mosquito nets prevent malaria								
No	116	6.11	50	8.26	66	5.10	7.19	0.007
Yes	1783	93.89	555	91.74	1228	94.90		
Malaria is curable								
No	91	4.79	49	8.10	42	3.25	21.30	< 0.001
Yes	1738	91.52	534	88.26	1204	93.04		
Don't know	70	3.69	22	3.64	48	3.71		
Able to list an antimalarial medicine								
No	410	23.59	89	16.67	321	26.66	20.50	< 0.001
Yes	1328	76.41	445	83.33	883	73.34		
Complete knowledge								
No	977	51.45	324	53.55	653	50.46	1.57	0.209
Yes	922	48.55	281	46.45	641	49.54		

### Characteristics of study participants by malaria knowledge level

Of the women interviewed, 48.6% correctly answered all five questions of interest about malaria. The difference in the level of knowledge about the causes, transmission, symptoms and treatment of malaria among women (Table 3) was statistically significant among the different age groups (p = 0.001), and among different levels of education, literacy, wealth quintiles, and in different languages used for interview (p < 0.001), as well as the number of births (p = 0.009), and television possession in the household (p = 0.008). No significant difference was noted by marital status (p = 0.958), place of residence (p = 0.209), radio possession in the household (p = 0.593), or exposure to a malaria message in the last six months (p = 0.593).

**Table 3:** Distribution of participants complete malaria knowledge according to sociodemographic characteristics, Mágoe District, 2019

Variables	Malaria complete knowledge		Total	χ^2^	p-value
	No n (%)	Yes n (%)			
	922 (48.55)	977 (51.45)	1899		
Group age					
15–19	269 (58.10)	194 (41.90)	463	22.8	0.001
20–24	218 (50.00)	218 (50.00)	436		
25–29	186 (51.81)	173 (48.19)	359		
30–34	117 (44.32)	147 (55.68)	264		
25–39	89 (43.63)	115 (56.37)	204		
40–44	69 (60.53)	45 (39.47)	114		
45–49	29 (49.14)	30 (50.85)	59		
Marital status					
Single	230 (51.34)	218 (48.66)	448	0.003	0.958
Married	747 (51.48)	704 (48.52)	1451		
Place of residence					
Mphende	324 (53.55)	281 (46.45)	605	1.6	0.209
Mucumburra	653 (50.46)	641 (49.54)	1294		
Level school					
None	221 (59.89)	148 (40.11)	369	33.1	< 0.001
Primary	401 (51.94)	371 (48.06)	772		
Primary complete	171 (50.89)	165 (49.11)	336		
Secondary	159 (48.04)	172 (51.96)	331		
Secondary complete or above	25 (27.47)	66 (72.53)	91		
Literacy					
Does not read	468 (58.87)	327 (41.13)	795	29.2	< 0.001
Read part of a sentence	223 (45.23)	270 (54.77)	493		
Read a whole sentence	284 (47.33)	316 (52.67)	600		
Number of births					
None	166 (59.50)	113 (40.50)	279	11.5	0.009
1 to 2 children	338 (50.15)	336 (49.85)	674		
3 to 4 children	272 (47.72)	298 (52.28)	570		
5 and more children	201 (53.46)	175 (46.54)	376		
Radio ownership					
No	664 (51.88)	616 (48.12)	1280	0.3	0.593
Yes	313 (50.57)	306 (49.43)	619		
Television ownership					
No	819 (52.91)	729 (47.09)	1548	7.1	0.008
Yes	158 (45.01)	193 (54.99)	351		
Exposure to malaria message in the last 6 months					
No	848 (51.64)	794 (48.36)	1642	0.2	0.593
Yes	129 (50.19)	128 (49.81)	257		
Wealth quintile					
Lowest	252 (58.88)	176 (41.12)	428	20.4	< 0.001
Second	176 (52.69)	158 (47.31)	334		
Medium	197 (48.28)	211 (51.72)	408		
Fourth	179 (53.27)	157 (46.73)	336		
Highest	173 (44.02)	220 (55.98)	393		
Language of interview					
Portuguese	37 (41.57)	52 (58.43)	89	26.5	< 0.001
English	2 (100.00)	0 (0.00)	2		
Cinyungwe	791 (49.91)	794 (50.09)	1585		
Ciyau	2 (100.00)	0 (0.00)	2		
Other	145 (65.61)	76 (34.39)	221		

### Factors associated with complete malaria knowledge

Table 4 presents the results of the models of factors associated with complete malaria knowledge. Compared with women aged 15–19 years, women in the age groups 30–34 (aOR = 1.8, 95% CI = 1.2–2.7) and 35–39 (aOR = 1.9, 95% CI = 1.1–3.1) had significantly higher odds of having complete malaria knowledge. Having completed secondary school or above (aOR = 2.5, 95% CI = 1.3–4.6), compared with women without any level of school education, and being able to read part of a sentence (aOR = 1.7, 95% CI = 1.3–2.1) were also associated with higher odds of complete malaria knowledge. The odds of having complete malaria knowledge were also significantly higher in women with 1–2 or 3–4 children (aOR = 1.8, 95% CI = 1.2–2.6, and aOR = 1.7, 95% CI = 1.0–2.6, respectively), as compared to those with none and in those who had their interviews completed in Portuguese or Cinyungwe (aOR = 2.3, 95% CI = 1.3–4.1, and aOR = 2.1, 95% CI = 1.5–2.8, respectively), as compared to those interviewed in an “other” language.

**Table 4:** Factors associated with malaria complete knowledge by women of reproductive age, Mágoe District, 2019

Variables	Bivariate		Multivariate	
	OR (IC95%)	p-value	aOR (IC95%)	p-value
Group age				
15–19	Ref	–	Ref	–
20–24	1.4 (1.1–1.8)	0.015	1.2 (0.9–1.6)	0.239
25–29	1.3 (0.9–1.7)	0.072	1.2 (0.8–1.7)	0.324
30–34	1.7 (1.3–2.4)	< 0.001	1.8 (1.2–2.7)	0.009
35–39	1.8 (1.3–2.5)	0.001	1.9 (1.2–3.1)	0.004
40–44	0.9 (0.6–1.4)	0.638	1.1 (0.6–1.9)	0.707
45–49	1.4 (0.8–2.5)	0.193	1.7 (0.9–3.1)	0.123
Marital status				
Single	Ref	–	–	–
Married	0.9 (0.8–1.2)	0.958	–	–
Place of residence				
Mphende	Ref	–	–	–
Mucumbura	1.1(0.9–1.4)	0.209	–	–
Level school				
None	Ref	–	Ref	–
Some primary	1.4 (1.1–1.8)	0.012	1.1 (0.8–1.4)	0.666
Primary complete	1.4 (1.1–1.9)	0.016	0.9 (0.7–1.4)	0.887
Some secondary	1.6 (1.2–2.2)	0.002	1.1 (0.7–1.8)	0.547
Secondary complete or above	3.9 (2.4–6.5)	< 0.001	2.5 (1.3–4.6)	0.005
Literacy				
Does not read	Ref	–	Ref	–
Read part of a sentence	1.7 (1.4–2.2)	< 0.001	1.7 (1.3–2.1)	< 0.001
Read a whole sentence	1.6 (1.3–2.0)	< 0.001	1.3 (0.9–1.8)	0.122
Number of births				
None	Ref	–	Ref	–
1 to 2 children	1.5 (1.1–1.9)	0.009	1.8 (1.2–2.6)	0.003
3 to 4 children	1.6 (1.2–2.1)	0.001	1.7 (1.0–2.6)	0.031
5 and more children	1.3 (0.9–1.8)	0.124	1.3 (0.8–2.1)	0.378
Radio ownership				
No	Ref	–	–	–
Yes	1.1 (0.9–1.3)	0.593	–	–
Television ownership				
No	Ref	–	Ref	–
Yes	1.4 (1.1–1.7)	0.008	0.9 (0.6–1.5)	0.792
Exposure to malaria message in the last 6 months				
No	1	–	Ref	–
Yes	1.1 (0.8–1.4)	0.665	0.9 (0.7–1.3)	0.733
Wealth quintile				
Lower	Ref	–	Ref	–
Second	1.3 (0.9–1.7)	0.088	1.2 (0.9–1.6)	0.253
Medium	1.5 (1.2–2.0)	0.002	1.5 (1.1–2.1)	0.005
Fourth	1.3 (0.9–1.7)	0.121	1.2 (0.9–1.7)	0.185
Highest	1.8 (1.4–2.4)	< 0.001	1.6 (0.9–2.7)	0.051
Language of interview				
Portuguese	2.7 (1.6–4.4)	< 0.001	2.3 (1.3–4.1)	0.004
English	1	–	1	–
Cinyungwe	1.9 (1.4–2.6)	< 0.001	2.1 (1.5–2.8)	< 0.001
Ciyau	1	–	1	–
Other	Ref	–	Ref	–

In this study, exposure to a malaria message in the last six months (aOR = 0.9, 95% CI = 0.7–1.3), and possession of television in the household (aOR = 0.9, 95% CI = 0.6–1.5) were not associated with complete knowledge about malaria.

## Discussion

This study assessed the factors associated with complete malaria knowledge among women of reproductive age in a rural Mozambique district and found that less than half of the women had complete knowledge. This low knowledge, along with low reported access to malaria information are potentially indicative of limited dissemination of malaria prevention measures by the health personnel or by the community health worker, which is not consistent with WHO recommendations or NMCP plans [1, 5]. Women are important in influencing malaria prevention practices within the family and community, and can contribute to the dissemination and implementation of prevention measures within households [17, 18] so this relatively low knowledge is problematic.

In this study, education level was shown to be associated with women's knowledge about malaria, as women with lower education levels had lower odds of having complete malaria knowledge. Women in Mozambique with low education are mostly rural residents, where access to health care and information is low and they may travel long distances to seek health care [19]. Women with higher levels of education tend to have more access to information, knowledge, and decision-making power about their health status and are mostly urban residents [20]. Studies in countries have shown similar results regarding the comparison of knowledge about malaria prevention in pregnant and non-pregnant women, and others have shown that the higher a woman's level of education, the greater the chance of having more access to information and having malaria knowledge [17, 21, 22]. These results underscore the importance of the broader educational and development context in malaria knowledge and behaviours and suggest that malaria prevention should be viewed in a transdisciplinary way.

This study also found that malaria knowledge was higher among women who spoke the national language or the dominant local language. It is a positive finding that information is reaching those who speak this dominant local language, but in a country such as Mozambique where there are over forty languages and only one official national language, Portuguese, this study shows that those who speak non-dominant local languages may represent a demographic with persistent information needs. Engagement with local leaders to better target and reach such women might be an effective strategy. For the wealth quintile, the middle quintile was associated with complete knowledge about malaria. This result was different from other studies, in which the highest wealth quintile was most associated with complete knowledge about malaria [18]. The influence of the wealth quintile not only depends on the availability of assets in the household but also on the relationship between the owner of those assets and the woman [23]. These differences may be due to the residence of the women, as this study was conducted in a predominantly rural area while the others compared rural and urban areas. Rural regions have the highest prevalence of malaria, and are the places where prevention activities must be most focused [6]. One important information source is during ANC service delivery which is generally accessed across the different socioeconomic groups and may explain this finding and the fact that women with more children were significantly more likely to have complete malaria knowledge [17, 24].

In the bivariate analysis, an association between the ownership of a television and knowledge about malaria was observed. Access to malaria information, through radio and television, has been shown to have good results in the implementation of malaria prevention measures, as seen in sub-Saharan African countries [25]. However, more in-depth studies could be conducted to find out which television channels are used the most in the location studied.

An interesting finding is the no association between hearing malaria message in the last six months and complete knowledge about malaria. The messages about malaria are disseminated through different strategies, in the ANC, through the community engagement, by community volunteers for dissemination of key messages about malaria [26]. Exposure to malaria messages, and their influence on the use of malaria prevention measures, is not always similar in studies [27]. In other studies, exposure to malaria messages to the community has been shown to be a positive factor in the implementation of malaria prevention measures [28]. This finding shows that malaria messages may not be being disseminated equally, or there may be a need to improve existing strategies.

## Limitations

This study design and inclusion criteria introduced a few limitations to the present analysis. Study inclusion was based on the existence of at least one child of five years of age or younger and at least one mosquito net per every two children in the household. These criteria meant that the population inherently had accessed a net in some way which may have contributed to their knowledge.

## Conclusions

In this rural setting in highly endemic Mozambique, complete malaria knowledge among women of reproductive age remained low and was significantly lower in more vulnerable populations, suggesting that efforts to disseminate malaria prevention key messages to women should involve more stakeholders to effectively target and implement SBC interventions. The lower access in younger women without children show the need for interventions to be targeted outside of ANC delivery such as in schools. Women of reproductive age are critical target populations for malaria prevention efforts and mapping and responding to these malaria information gaps can help improve individual and familial health.

## Data Availability

The datasets for this study are available upon a reasonable request.

## Abbreviations

ANC: Antenatal Care
aOR: Adjusted Odds Ratio
CDC: Centers for Disease Control and Prevention
CI: Confidence Interval
EA: Enumeration Areas
IPTp-SP: Intermittent Preventive Treatment in Pregnancy with Sulfadoxine-Pyrimethamine
IQR: Inter-Quartile Range
IRS: Indoor Residual Spraying
ITN: Insecticide-Treated Net
MIS: Malaria Indicators Survey
OR: Odds Ratios
SBC: Social and Behaviour Change
WHO: World Health Organization
